# Development of Technologies for Processing Polypropylene Foil Waste and Their Use in the Production of Finished Products

**DOI:** 10.3390/ma17215192

**Published:** 2024-10-24

**Authors:** Damian Dziadowiec, Karina Walburg, Danuta Matykiewicz, Jacek Andrzejewski, Marek Szostak

**Affiliations:** Faculty of Mechanical Engineering, Poznan University of Technology, Piotrowo 3, 61-138 Poznan, Poland; karina.walburg@student.put.poznan.pl (K.W.); jacek.andrzejewski@put.poznan.pl (J.A.); marek.szostak@put.poznan.pl (M.S.)

**Keywords:** polypropylene, cast foil waste, injection, extrusion

## Abstract

This work aims to assess the possibility of using packaging industry waste to modify polypropylene products (PPs). The products were made in the form of extruded foil and injected samples. The products were produced using regranulate made of polypropylene cast foil. Maleic anhydride-modified polypropylene (MAPP) and polyolefin elastomer (POE) with a glycidyl ester functional group were used to modify the polypropylene. The samples were produced based on 50% foil waste reground and 50% pure PP. The rheological properties of the blends were assessed using the melt mass flow rate (MFR) technique; thermal properties using the differential scanning calorimetry method (DSC). The products manufactured using the injection molding method were subjected to an analysis of mechanical properties, such as tensile strength and impact strength. Also, in the case of film samples, tensile strength was assessed. Color-change assessments with CIE L*a*b* were carried out for all materials. Injection-molded products based on recycled metallized cast foil showed favorable mechanical properties such as tensile strength (1 MAPP = 26.7 MPa; 2 MAPP = 27.1 MPa), which was higher than the original material (cPP = 20.7 MPa). Also, for the films produced from regrind, the tensile strength was at a level similar (1 MAPP = 24.6 MPa; 2 MAPP/POE = 25.1 MPa) to the films extruded from virgin materials (cPP = 24.9 MPa). The introduction of a POE additive to the blends resulted in increased impact strength (1 MAPP/POE = 31 kJ/mol; 2MAPP/POE = 18 kJ/mol; 3 MAPP/POE = 11 kJ/mol) in relation to unmodified samples (cPP = 7 kJ/mol). The introduction of a POE additive to the tested mixtures improved the impact strength of the injected products by almost 4 times for sample 1 MAPP/POE and 2.5 times for sample 2 MAPP/POE in comparison to virgin cPP. These studies confirmed that foil waste can be successfully used to modify polypropylene products shaped both in the injection and extrusion processes.

## 1. Introduction

Due to the increasing consumption of packaged food and consumer goods made of plastic, a large amount of waste remains on the market after their use. Developing an effective method for the selection, collection, and industrial processing of waste generated from polymeric materials is still a current research topic worldwide [1,2]. Polyolefins are widely used due to their ease of processing, average price, favorable mechanical properties, and resistance to chemical compounds [3]. Recycled post-consumer waste is described as PCR. Products based on these can be successfully used in households or industrial applications. Groups of materials such as polyethylene, polypropylene, or polyethylene terephthalate can be easily recycled, significantly reducing the consumption of non-renewable raw materials [4]. According to data, the use of recyclates has increased by 70% since 2018, and circular plastics currently constitute 13.5% of all plastics used by European processors to produce new plastic products and parts [5]. Unfortunately, it is estimated that around 80% of the estimated total plastic produced, i.e., 6.3 trillion tons of plastic, is thrown away [6]. This is a massive loss of resources, confirming the mismanagement of waste and a growing ecological disaster. To prevent the loss of material resources and environmental pollution, plastic management should be implemented by the principles of a circular economy [7]. Depending on the origin of the material, its structure, and composition, an appropriate material recovery method can be made [8].

The applications of polymer films can be divided into packaging and non-packaging. Packaging is divided into consumer (primary packaging) and non-consumer (secondary and tertiary packaging) [9]. According to Plastics Europe, 18.5 Mt of post-consumer plastic packaging waste was managed in Europe in 2022, of which 17.3% went to landfills, 37.8% was recycled, and 44.9% was used for energy recovery [10]. Recycling packaging is often more economically viable than other sectors of the plastics market [11,12]. Recycling rates for plastic packaging waste are 60% higher when collected separately, compared to mixed collection streams. For comparison, the management of post-consumer plastic construction waste (2.3 Mt) is as follows: 30.4% is landfilled, 17.3% is recycled and 52.2% is subject to energy recovery. Similar data were recorded for post-consumer electrical and electronics plastic waste, where 30% went to landfills, 20% was recycled, and 50% was recovered for energy [10]. Extrusion is an efficient, relatively cheap and safe method used in mechanical recycling to produce regranulate from typical plastic waste [13]. Due to the chemical and physical forces acting during extrusion, mechanical recycling often reduces the tensile strength and elongation at the break of polypropylene, polyethylene, and polyethylene terephthalate materials [14,15]. To eliminate unfavorable phenomena occurring during processing, various types of modifiers are used, including stabilizers [16], antioxidants [17], impact modifiers [18], compatibilizers [19], and plasticizers [20].

Polypropylene is one of the most commonly used thermoplastic polymers due to its low price and beneficial properties [21]. PP-based films are produced by extrusion as cast films and oriented films. Cast films are made by depositing a layer of plasticized polymer on the surface and solidifying it by cooling the melt or evaporating the solvent. This allows for the obtaining of flexible and elastic packaging materials. To improve a PP’s poor oxygen barrier properties, it is combined with other materials to create layered films with specific features [22,23]. Coating polypropylene foils with a layer of aluminum is commonly used in food packaging because aluminum is an excellent barrier to gasses. However, recycling these foils is difficult and economically unprofitable [24]. Riedewald et al. presented a method for recycling aluminum packaging using a molten metal pyrolysis reactor to recover pure aluminum [25]. Seier et al. described the prospects for the mechanical recycling of commercial, post-consumer PET and PP packaging used for modified atmosphere packaging. Due to PET products’ poor mechanical properties and difficult processability, the multiple processing of this waste stream was impossible. However, PP-based packaging (lid + tray) was recycled ten times to demonstrate the recyclability [26]. Guerritore et al. demonstrated easy recyclability of the coated films with high gas and UV barrier properties by using graphene oxide (GO) and graphene oxide/montmorillonite (GO/MMT) hybrid coatings on polyethylene and polypropylene cast foils [27]. Also, a commonly explored method is solvent-based processes to recycle multilayer films. Multilayer packaging often contains adhesives, including acrylic and polyurethane, to join different materials [28]. The decomposition or dissolution of the binder layer from the multilayer structure allows the target materials to be separated while maintaining their original shape and structure [29]. Developing appropriate methods to safely process multi-component wastes continues to challenge researchers and manufacturers [30]. It should be emphasized that due to the production of an increasing number of plastic packaging with various compositions, functionalities, and shapes, selecting appropriate methods for processing the waste generated in this way is still the subject of research and implementation work by many scientists and manufacturers. However, the non-polar nature of polypropylene is a disadvantage in applications requiring compatibility and adhesion with polar molecules. Therefore, isotactic polypropylene (PP) is grafted with maleic acid (MA), which is useful as a compatibilizer for blends with various ingredients [31]. Coupling agents contribute to the increased interfacial stress required to debond the filler/polymer interface, which should provide higher tensile and flexural strength and increased stiffness [32]. On the other hand, POE is a double functional group containing a glycidyl ester-grafted polymer and can improve impact property and bonding strength between different components [33]. Due to the problems of film cracking during the packaging operation of the products, an impact modifier such as POE acts as an agent improving the flexibility of the polymer film and, at the same time, its impact strength [34]. In addition, the functional groups present in the POE modifier act as a coupling agent [35].

The challenges associated with the production of multilayer films primarily lie in ensuring good connections between all layers, as well as appropriate mechanical properties, such as resistance to cracking or strength, which, in the context of using these products as packaging, is of great importance for their use for a specific type of product.

Therefore, this work aimed to demonstrate methods of managing multilayer foil waste by reprocessing it into finished products. Maleic anhydride-modified polypropylene (MAPP) and polyolefin elastomer (POE) with a glycidyl ester functional group were used to improve blend uniformity. Samples were produced from 50% recycled foil and 50% of an origin PP. The rheological properties of the blends were assessed using the melt mass flow rate (MFR) technique, and thermal properties were evaluated using the differential scanning calorimetry method. The products manufactured using the injection molding method were subjected to analysis of mechanical properties, such as tensile strength and impact strength. Also, in the case of film samples, tensile strength was assessed. Color change assessment was carried out for all materials.

## 2. Materials and Methods

### 2.1. Materials and Sample Preparation

The PP random copolymer (cPP) RD234CF (Borealis, Vienna, Austria) was used as a base for manufacturing blends with waste foils. Three types of waste multilayer foils were used to produce the samples:

Foil 1—A three-layer A/B/C foil consisting of layer A, a PP copolymer sputtered with aluminum; layer B, a PP homopolymer; and layer C, a PP copolymer; thickness: 50 µm.
Foil 2—A laminate consisting of one layer of biaxially oriented polypropylene (BOPP) foil sputtered with aluminum with a three-layer foil A/B/C: layer A—PP homopolymer with additives; layer B—homopolymer with and without additives; layer C—cPP copolymer with additives; thickness: 70 µm.
Foil 3—A laminate consisting of one layer of biaxially oriented poly(ethylene terephthalate) (BOPET) foil sputtered with aluminum combined with a three-layer foil A/B/C: layer A—PP homopolymer with additives; layer B—PP homopolymer with additives; layer C—cPP copolymer; thickness: 90 µm.

**Figure 1:**
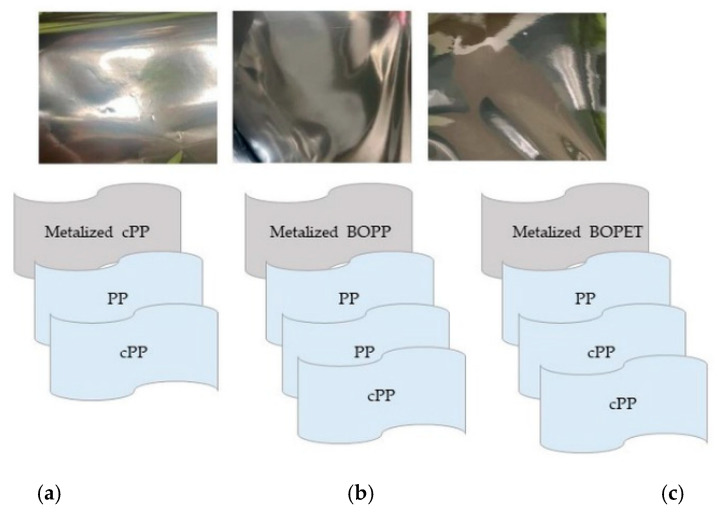
Three different types of foil waste: (**a**) Foil 1, (**b**) Foil 2, (**c**) Foil 3; PP- polypropylene homopolymer; cPP- polypropylene copolymer.

**Figure 2:**
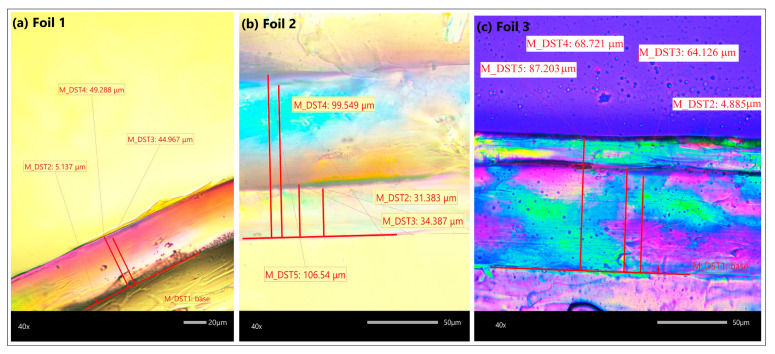
Microscopic photos of foil waste: (**a**) Foil 1, (**b**) Foil 2, (**c**) Foil 3.

All materials also contained glue between the layers used to make this laminated film. The appearance of the foil is shown in Figure 1 and Figure 2. All of the multilayer film waste was supplied by Eurocast company (Strzebielino, Poland), and the material is post-production waste generated during the start-up of mass production on the extrusion line. The following modifiers produced by Fine-Blend^®^ (Shanghai, China) were used: maleic anhydride-modified polypropylene CMG5701 (MAPP) as a coupling agent and polyolefin elastomer (POE) SOG-02 with a glycidyl ester functional group. The MAPP additive improves adhesion between the PP and polar substrates such as glass, metal, and minerals. As an elastomer with high flexibility, POE is used to enhance the impact property of the material. The material composition of each sample is summarized in Table 1.

**Figure 3:**
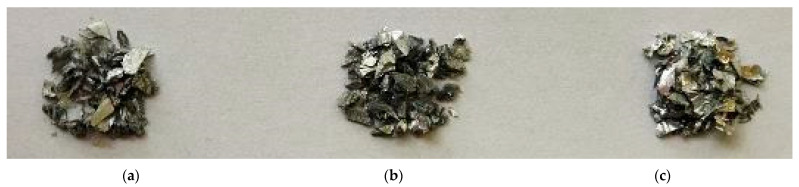
Three different types of regrind: (**a**) Foil 1, (**b**) Foil 2, (**c**) Foil 3.

**Scheme 1:**
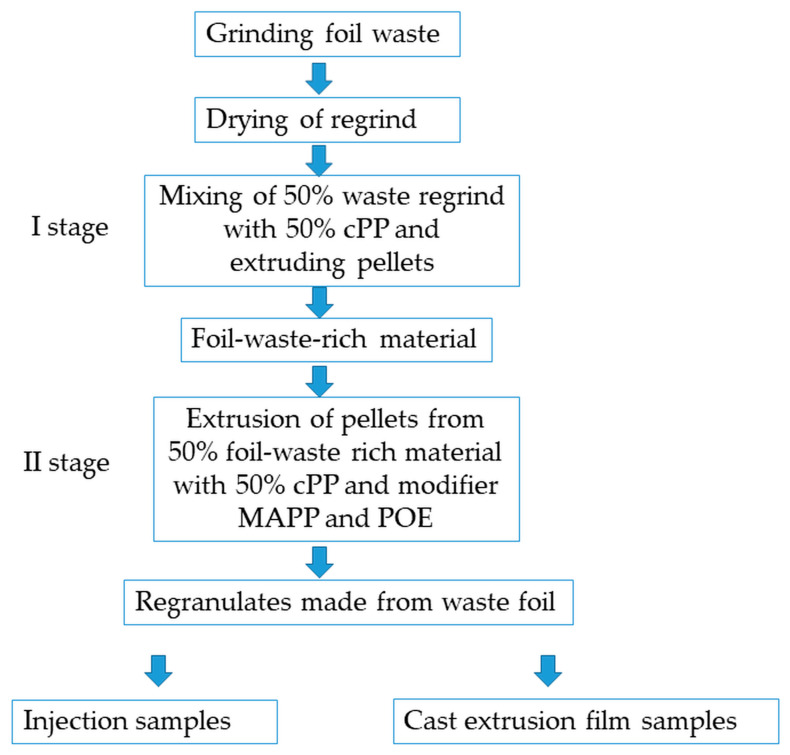
Method of sample preparation.

**Figure 4:**
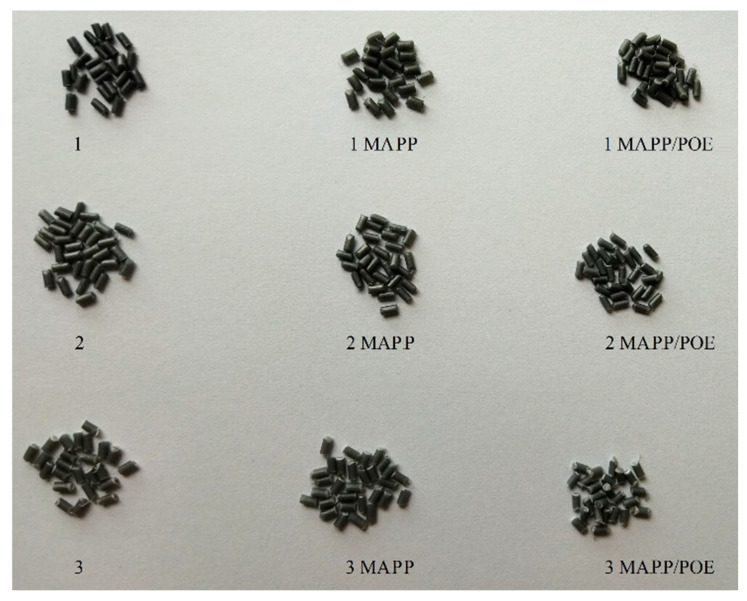
Regranulates made from waste foil.

The waste foil was ground using a low-speed granulator GV 270 Virginio Nastri SLR (Gambellara, Italy) with a 5 mm granulator sieve, and the resulting regrind was used to produce a new product. The appearance of the obtained regrind is shown in Figure 3. The regrind obtained in this way was dried at 80 °C for 4 h. The sample preparation method is presented in Scheme 1. In the first step, the regrind was mixed with pure cPP in the proportions of 50% + 50% by mass to obtain a material rich in foil waste, followed by melt mixing. The first-stage compound pellets were blended with cPP and additives (MAPP and POE). For both blending stages, the prepared mixtures were subjected to extrusion using a twin-screw extruder, model Zamak EH16.2D with L/D 30 (Zamak Mercator, Skawina, Poland), to obtain pellets. The extruder barrel was heated to 230 °C for PP-based foil and 260 °C for PET-based foil; after melting and mixing, the material was cooled and pelletized. All types of blends were prepared at the same conditions, with the following barrel temperature: 230(260)−230(260)−230(260)−220–220−210–200−200–190 °C. The screw speed was set to 120 rpm. We used the same belt conveyor for extrudate cooling, and knife pelletized for granulate preparation. It should be emphasized that the regranulate (pellets) produced on the basis of ground waste foils was used to manufacture two types of products: injected parts and extruded foils. The appearance of the obtained regranulates is shown in Figure 4. Due to the presence of the metal layer, all materials were characterized by a gray shade.

The injection molding process was performed by the Engel Victory 50 hydraulic machine (Engel Austria GmbH, Schwertberg, Austria) with a 25 mm screw and 500 kN molding press. The shapes were produced with the following process parameters: molding temperature = 260 °C, mold temperature = 20 °C, injection pressure = 1000 bar, holding pressure = 500 bar, holding time = 6 s, and cooling time = 35 s. During processing, the material was shaped into the form of dumbbell samples (1A—ISO 527 standard) [34] and sample bars (10 × 10 × 80 mm).

A single-screw extruder manufactured the foil products, Metalchem W25–30D (IMPiB, Torun, Poland), equipped with a flat die and chill roll system. The processing parameters were as follows: extruder barrel temperature = 220(260) °C, die temperature = 230(270) °C, screw speed = 30 rpm, take-up roller temperature = 40 °C (values in brackets refer to blends containing Foil 3). All foil samples were extruded at single draw speeds of 5 m/min.

### 2.2. Methods

The photographs of the foil were taken using a Leica DM2500 optical microscope (Leica, Wetzlar, Germany). The number of repetitions was 3.

The investigated mixtures of polyamide with regrind were measured at the melt mass flow rate (MFR) using a Dynisco 4004 melt flow indexer (Dynisco, Franklin, MA, USA) according to ISO 1133 (load 2.16 kg, temperature 230 °C) [36].

Differential scanning calorimetry (DSC) analysis was conducted using a Netzsch DSC 204F1 Phoenix (Selb, Germany). Samples weighing 5.0 ± 0.1 mg were placed in aluminum crucibles with pierced lids, heated in the 25–300 °C range and cooled in the 300–25 °C range at a rate of 10 °C/min under an inert nitrogen atmosphere with two heating/cooling cycles.

The tensile test of the injection-molded samples was carried out on a Zwick/Roell Z010 universal testing machine (Zwick, Germany) in accordance with the ISO 527 standard [37]. We used 1A-type specimens at a head speed of 50 mm/min. Samples of 15 mm width were cut from the extruded foils and stretched by the ISO 527 standard at a 200 mm/min speed.

The impact strength of the rectangular notched samples 80 × 10 × 4 mm was assessed by the Charpy method (ISO 179) at room temperature, using a Zwick/Roell HIT 25P impact strength apparatus (Zwick, Germany) with a 5 J hammer [38].

Color changes were assessed by determining the L*a*b chromatic parameters, according to the International Commission on Illumination (CIE). The analysis was performed by portable spectrophotometer—NR145 Precision Colorimeter 3nh (Shenzhen, China) equipped with standard light illuminant D65 and aperture F8. In this color space, L* is the color lightness (L* = 0 for black and L* = 100 for white), a* is the green (−) to red (+) axis, and b* is the blue (−) to yellow (+) axis [39].

## 3. Results

### 3.1. Melt Flow Rate

MFR measures the flow of a melted thermoplastic under the influence of applied stress. It is defined as the mass of plastic flowing through a capillary matrix of specified diameter and length under a specified load and temperature. This parameter directly impacts the selection of processing parameters during the process, determining the ease of flow of the polymer in the melted state. Thermoplastic polymers with a low MFR (e.g., cPP RD234CF = 8 g/10 min, Borealis) are used as raw materials during extrusion, and polymers with a higher MFR value are used for injection molding (e.g., BH374MO = 40 g/10 min, Borealis). For example, the commercial black polypropylene PP-04 (Borealis) obtained from post-industrial recyclates is characterized by an MFR in the range of 2–15 g/10 min. MFR according to the manufacturer for MAPP is 50–100 g/10 min and for POE is 2–5 g/10 min. It can be seen that the addition of both MAPP and POE reduced the MFR value of the tested materials (Table 2). It can be assumed that the reduction in the MFR of blends with the addition of MAPP results from the introduction of a branched structure into the polymer matrix, which increases the mobility between the chains. MFR describes the fluidity of polymer melts. Analyzing the data from Table 2, it can be seen that the fluidity of the blends decreased with the addition of POE. This may be due to the increased entanglement between molecular chains due to the addition of POE with regular short and long side chains, which affects the relative mobility of the molecules [40]. Determining the MFR value is often crucial in determining the potential of recycled waste applications and their reuse within the circular economy.

### 3.2. Differential Scanning Calorimetry

**Figure 5:**
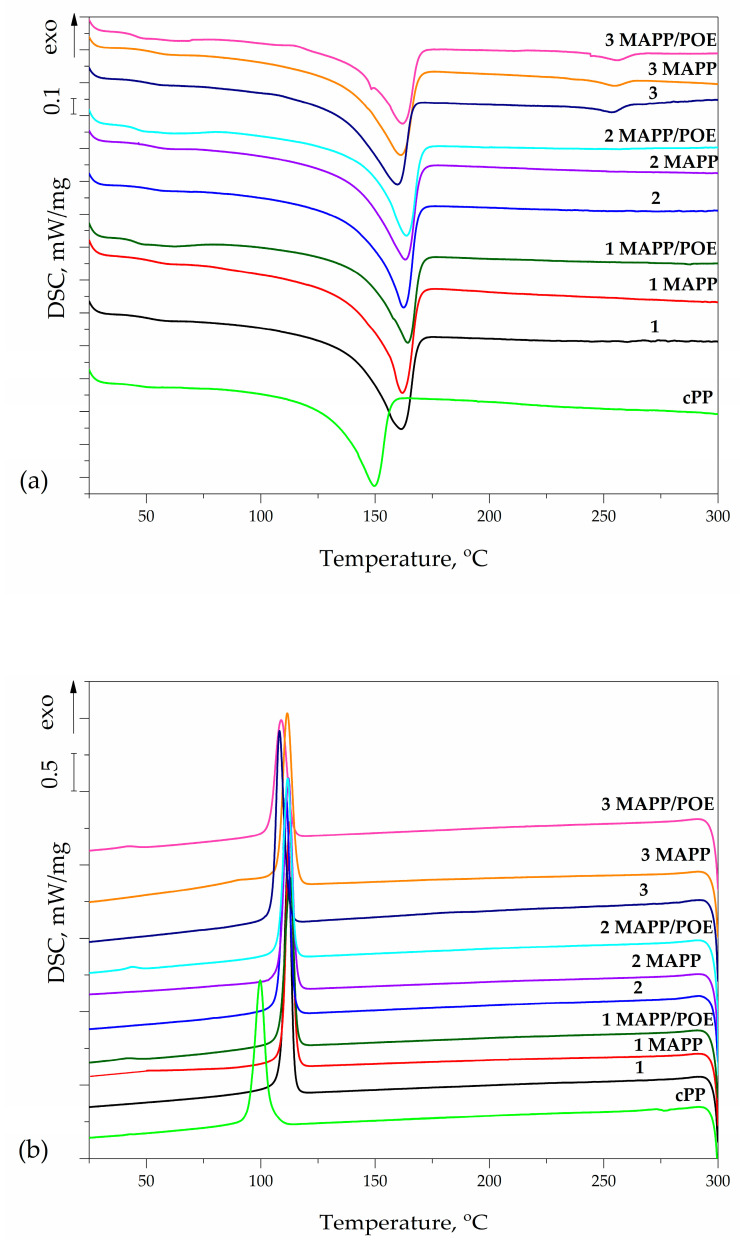
DSC thermographs of the samples: (**a**) first heating cycle; (**b**) first cooling cycle.

As a result of differential scanning calorimetry measurements, parameters such as melting temperature (Tm), crystallization temperature (Tc), and melting enthalpy (Hm) were determined. The DSC curves recorded during the first heating and first cooling cycles are shown in Figure 5. The melting temperatures from the first and second heating (Tm_I_ and Tm_II_), the crystallization temperature Tc from the first cooling cycle, and the melting enthalpy of fusion (ΔHm_I_ and ΔHm_II_) are summarized in Table 3. Due to the significant portion of polypropylene in the blends, one dominant melting peak is visible on the DSC curves. The melting temperature of the tested blends determined from the first heating cycle was averaged from 160 to 163 °C. The thermal properties were slightly improved by adding MAPP and POE modifier, as the first melting occurs at higher temperatures than in the case of unmodified film-based materials [41]. The crystallization temperature of all tested mixtures was similar and amounted to approx. 111 °C, except for mixtures based on a film containing PET, where a slight decrease in Tc to 108 °C was observed. These changes may result from the inappropriate combination of ingredients in the three foil-based mixtures and their inhomogeneous structure [42].

### 3.3. Tensile Strength

The possibility of using recycled materials depends mainly on the impact of their addition on the mechanical properties and usability of finished products [43]. A significant deterioration of these parameters limits the use of recyclates in their original range but makes it possible to find areas where lower properties are acceptable [44]. Table 4 summarizes the mechanical properties determined for the injected samples. The tensile modulus, tensile strength, and elongation at break were determined during the tensile test. The highest elasticity modulus values were recorded for injection-molded parts based on films 1 and 2: 1 MAPP/POE (1100 MPa) and 2 MAPP (1110 MPa). This phenomenon may indicate an increased stiffness of the tested samples obtained due to their modification. It should also be emphasized that the introduction of MAPP as a coupling agent into the blends improved the homogeneity of the different component materials, such as polymer and metal, which resulted in increased tensile modulus values (especially for 1 MAPP; 1 MAPP/POE and 2 MAPP) compared to the unmodified materials.

The tensile strength values were similar for samples based on films 1 and 2, averaging 26.0 MPa, except for compositions 1 MAPP/POE and 2 MAPP/POE. The combination of two types of modifiers did not result in the expected enhancement of the strength properties of the tested materials. The lowest tensile strength values were recorded for cPP (20.7 MPa), 3 MAPP/POE (19.6 MPa) and 2 MAPP/POE (20.3 MPA), which were 23%, 27%, and 25% lower, respectively, compared to the highest value recorded for 2 MAPP (27.1 MPa). Among the entire group of tested materials, it can be seen that samples based on film 3 containing two types of polymers, PP and PET, exhibit lower strength properties in comparison to products made of one type of polymer, i.e., PP. The effect of mechanical recycling on the properties of injection-molded polypropylene (PP) reinforced with thermotropic liquid crystalline polymer (TLCP) or long glass fiber (GF) was described by Chen et al. They proved that the addition of MAPP increases the tensile modulus by 15% and the tensile strength by 21% as compared to the incompatibilized 50 wt% TLCP/PP [45]. Likewise, in this work, an increase in tensile modulus is observed for samples containing MAPP. Velásquez et al. also proved that adding MAPP to mixtures of post-industrial recycled polypropylene (PIPP) with nanoclay improves the compatibility of ingredients [46]. The similarity of the MAPP backbone and the PP structure promoted interaction between the compatilizer and the PP matrix as well as the other components, as the results showed that an improvement mechanical properties was achieved [47]. Behazin et al. also observed the beneficial effect of using MAPP to improve the properties of polypropylene composites [48].

The tensile test results carried out for the films produced from waste are collected and displayed in Table 5. The highest elasticity modulus values were recorded for extruded films based on waste foil, PP, and BOPP for sample 2 (227 MPa) and 2 MAPP/POE (158 MPa). This phenomenon may indicate an increased stiffness due to the effective modification and homogeneity of these blends. Also, for this group of films, the highest tensile strength values were recorded (on average 25 MPa), comparable to pure cPP film (24.9 MPa). For films extruded from a dual-material regranulate containing PP and BOPET, the values of tensile strength modulus and elongation values were the lowest. This indicates low homogeneity and mixing of components in this blend. A dramatic decrease in elongation was also observed for the sample 3 MAPP. For this series of extruded films, both MAPP and POE modifications produced the desired effect of increasing the strength and elasticity modulus of the film. The very low elongation value a of the sample is due to its inhomogeneity and considerable brittleness, resulting from poor bonding of the mixture components [49,50].

### 3.4. Impact Strength

**Figure 6:**
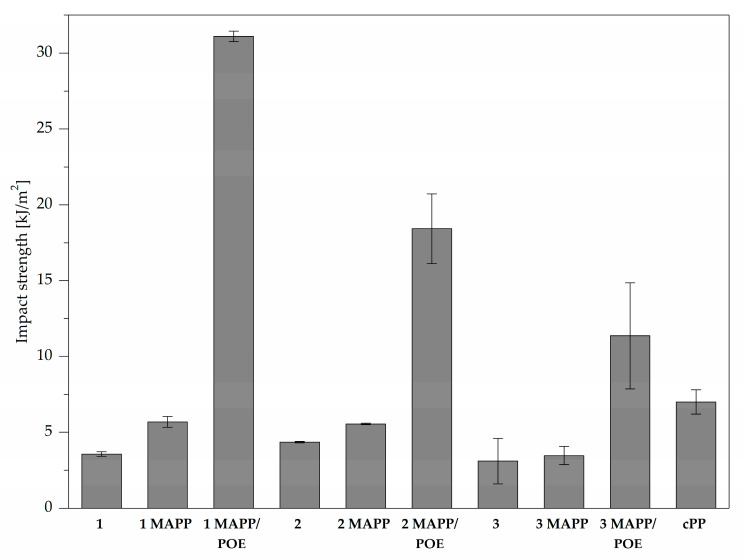
Impact strength of the tested injection-molded samples.

Table 4 and the graph summarize the impact strength results for the tested materials. In the case of samples containing POE, an increase in impact strength can be observed compared to unmodified samples. For samples, 1 MAPP/POE and 2 MAPP/POE, the highest impact strength values of 31 and 18 kJ/m^2^, respectively, were recorded. Polyolefin elastomers are compatible with most olefin materials and are an excellent impact modifier for plastics. Yang et al. described [51] that the addition of POE to the PP significantly improved the impact strength of the blends even at low temperatures. Luo et al. also confirmed the beneficial effect of this additive on the impact strength of polypropylene [52]. The introduction of a POE additive to the tested mixtures improved the impact strength of the injected products almost 4 times for sample 1 MAPP/POE and 2.5 times for sample 2 MAPP/POE in comparison to pure cPP. Among the entire group of tested materials, it can be seen that for samples based on Foil 3, the increase in impact strength was not significant after modification. It can also be observed that the presence of aluminum particles improved the impact strength of blends concerning pure cPP for sample 3 MAPP/POE (11.36 kJ/m^2^). The summary of the impact strength of the tested injection-molded samples is presented in Figure 6.

### 3.5. Color Changes

**Figure 7:**
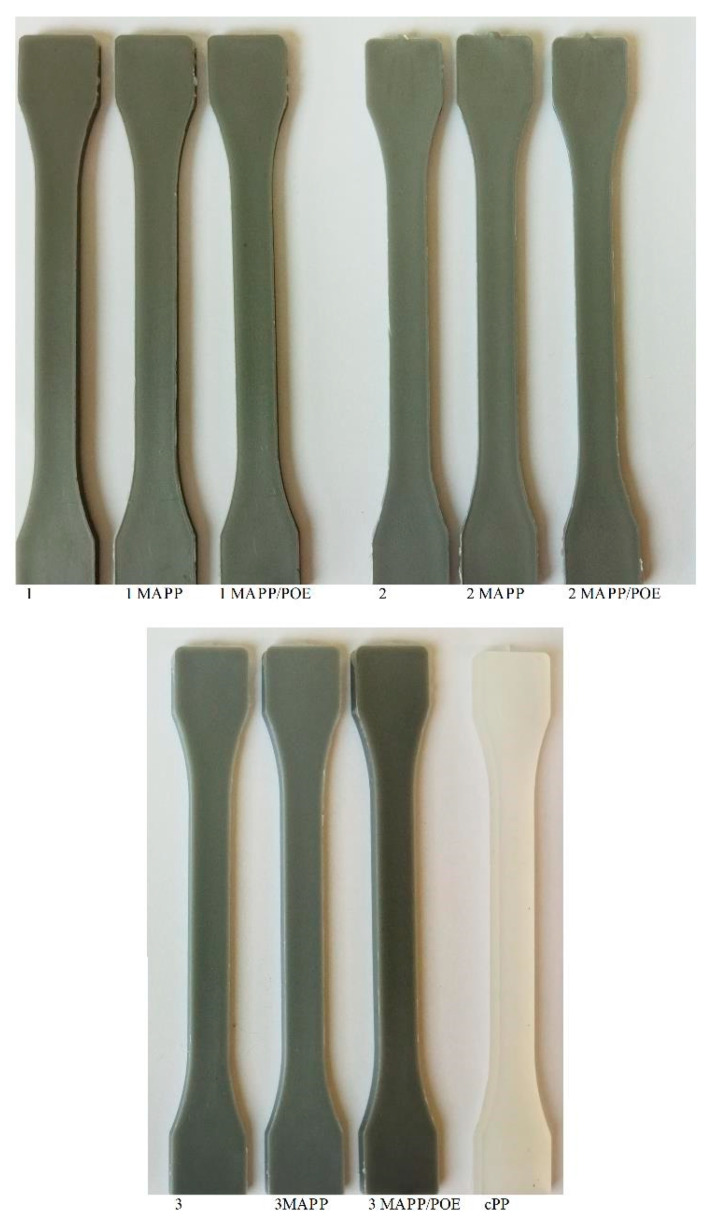
The color of the tested injection-molded samples.

**Figure 8:**
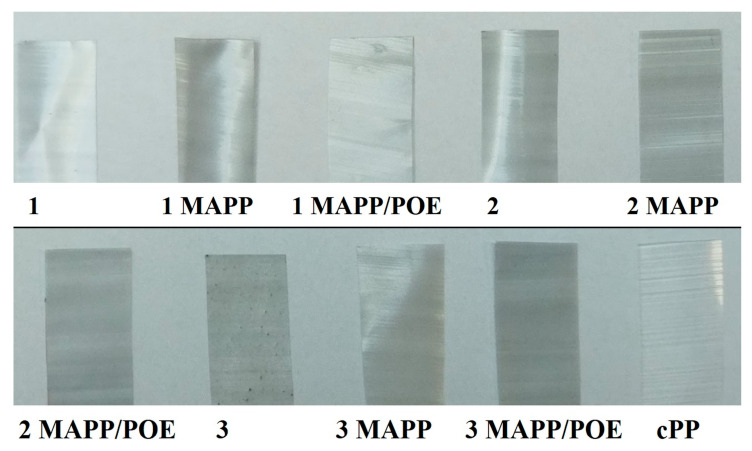
The color of the tested foil samples.

The CIE L*a*b color test is commonly used to assess the color of a product after mechanical recycling processes [53] or after degradation under controlled conditions [54]. Table 6 and Table 7 summarize the data obtained during the color change measurements expressed by CIE L*a*b* coordinates—the appearance of the tested injection-molded shapes is presented in Figure 7. The best optical properties are characteristics of the sample made of pure polypropylene. All samples made from regranulates have a dark gray color. This is due to the sputtered aluminum layer on the foils used. Based on Foil 3, waste samples containing BOPET and PP have the most diverse color than other samples using regranulates. This is due to the lack of homogenization between PET and PP. No significant color differences were observed between samples produced from Foil 1 and based on PP, and PP and BOPP, regardless of the recipe composition. The photos of the tested foils based on waste materials are shown in Figure 8.

## 4. Discussion

Plastic recycling can be described as the process of valorizing old-component waste or post-consumer plastic products and reprocessing them into products of full value [55]. The growing consumption of individually packaged food and consumer goods brings enormous amounts of foil packaging that, after their life cycle, end up in recycling plants or landfills. Illustrating a means of using waste materials containing different types of polymers and other layers is valuable information for future manufacturers. It is estimated that in developed countries, around 50% of plastics in household waste are foils [56]. Thermoplastics used as packaging materials have a life cycle of less than one, unlike polymeric materials used in the automotive, electronics and construction industries [57]. In tertiary and secondary packaging, the monolayer film consists of a thermoplastic polymer sheet made of PE, PP or PET, the thickness of which is usually in the range of 20 to 200 µm [56]. In the case of multilayers, the most important application is the production of primary packaging. Multilayer packaging for food or other goods can be divided into flexible or semi-rigid packaging, containing polymeric or inorganic layers [58]. Each layer fulfills a specific function in the entire packaging system. The most commonly used plastics in these particular products are polyolefins, including polypropylene and polyethylene, mainly due to their low price and appropriate stability, flexibility, strength, and chemical resistance. The use of aluminum foil or a metalized aluminum layer on PP, PE, or PET polymer foil allows for the improvement of the barrier properties of the packaging. The most commonly used laminates with the addition of Al are biaxially oriented polyethylene terephthalate (BOPET) and biaxially oriented polypropylene (BOPP) [59]. Based on the conducted research, it can be stated that metalized foil waste is suitable for reprocessing after appropriate preparatory treatment such as grinding and regranulation. This feedstock could be used for the production of products for which lower properties are acceptable, e.g., flowerpots or gardening foils. In recent years, large-scale injection-molded components in the automotive industry, such as cockpit elements or exterior housings, have been manufactured from recycled plastics. The amount of polymer plastic recovered for reuse is increasing year by year. For example, 680 tons of polypropylenes were recycled by French ELV processing centers in 2015, which is equivalent to the bumpers and wheel arches of 42,000 vehicles [60].

## 5. Conclusions

The conducted research confirmed that the grinding and regranulation technology is an effective method of recovering polymer material from foil waste, which can be successfully used to produce shaped products using both injection and extrusion methods. The obtained results proved that the introduction of two types of modifiers, i.e., maleic anhydride-modified polypropylene (MAPP) and polyolefin elastomer (POE) with a glycidyl ester functional group, to blends enabled the production of homogeneous polymer blends. The products based on waste Foil 1 and Foil 2 modified with MAPP and POE were characterized by improved mechanical properties such as the impact and tensile strength of injected polypropylene products. Injection-molded products based on recycled metalize cast foil showed favorable mechanical properties such as tensile strength (1 MAPP = 26.7 MPa; 2 MAPP = 27.1 MPa) that is higher than the original material (cPP = 20.7 MPa). Also, for the films produced from regrind, the tensile strength was at a level similar (1MAPP = 24.6 MPa; 2MAPP/POE = 25.1 MPa) to the films extruded from virgin materials (cPP = 24.9 MPa). A clear trend of increasing impact strength of the injected samples can be observed for samples containing POE elastomer (1MAPP/POE= 31 kJ/mol; 2 MAPP/POE = 18 kJ/mol; 3 MAPP/POE= 11 kJ/mol) in relation to unmodified samples (cPP = 7 kJ/mol). To sum up, foil waste can be used to produce new materials. The selection of this appropriate modification and processing method is a response to the market demand for products manufactured from recycled raw materials.

## Figures and Tables

**Table 1:** Material composition of individual samples.

Name	Type of Waste Foil	Recyclate Content [%]	cPP [%]	MAPP [%]	POE [%]	Thickness [µm]
1	Foil 1	25	75	0	0	150 ± 9
1 MAPP	Foil 1	23.75	71.25	5	0	150 ± 7
1 MAPP/POE	Foil 1	20	60	5	15	145 ± 5
2	Foil 2	25	75	0	0	125 ± 5
2 MAPP	Foil 2	23.75	71.25	5	0	153 ± 3
2 MAPP/POE	Foil 2	20	60	5	15	140 ± 5
3	Foil 3	25	75	0	0	160 ± 5
3 MAPP	Foil 3	23.75	71.25	5	0	100 ± 5
3 MAPP/POE	Foil 3	20	60	5	15	155 ± 5
cPP	-	0	100	0	0	155 ± 5

**Table 2:** Melt flow rate of regranulates obtained from foil waste.

Name	MFR [g/10 min]
1	10.4 ± 0.5
1 MAPP	6.9 ± 0.2
1 MAPP/POE	2.3 ± 0.1
2	8.7 ± 1.4
2 MAPP	7.3 ± 0.4
2 MAPP/POE	5.7 ± 0.2
3	5.8 ± 0.2
3 MAPP	4.8 ± 0.1
3 MAPP/POE	3.4 ± 0.1
cPP	8.0 ± 0.1

**Table 3:** Melting Tm_I_ and Tm_II_, crystallization temperatures Tc, and melting enthalpy of the investigated regranulates.

Name	Tm_I_ (°C)	ΔHm_I_ (J/g)	Tc_I_ (°C)	Tm_II_ (°C)	ΔHm_II_ (J/g)
1	161.4 ± 0.2	74.1 ± 0.2	111.9 ± 0.2	154.0 ± 0.1	79.2 ± 0.1
1 MAPP	162.0 ± 0.1	76.3 ± 0.1	112.5 ± 0.3	154.1 ± 0.2	90.4 ± 0.2
1 MAPP/POE	164.3 ± 0.3	61.8 ± 0.2	112.8 ± 0.1	158.0 ± 0.4	69.8 ± 0.3
2	162.5 ± 0.2	74.3 ± 0.3	111.1 ± 0.2	153.1 ± 0.3	79.6 ± 0.1
2 MAPP	163.3 ± 0.1	75.9 ± 0.2	112.1 ± 0.4	154.7 ± 0.3	82.6 ± 0.3
2 MAPP/POE	163.8 ± 0.4	65.1 ± 0.3	111.8 ± 0.3	156.3 ± 0.2	67.5 ± 0.2
3	159.7 ± 0.4	67.4 ± 0.1	108.2 ± 0.4	151.5 ± 0.7	71.1 ± 0.1
3 MAPP	161.2 ± 0.5	70.1 ± 0.4	111.6 ± 0.5	153.4 ± 0.6	98.2 ± 0.1
3 MAPP/POE	162.2 ± 0.5	59.1 ± 0.2	108.9 ± 0.6	157.1 ± 0.7	59.7 ± 0.2
cPP	149.6 ± 0.1	60.0 ± 0.2	99.7 ± 0.2	143.2 ± 0.2	65.1 ± 0.3

**Table 4:** Mechanical properties of injection-molded samples based on foil recyclate.

Name	Tensile Modulus [MPa]	Tensile Strength [MPa]	Elongation at Break [%]	Impact Strength (Notched) [kJ/m^2^]
1	984 ± 24.5	26.1 ± 0.5	9.5 ± 0.2	3.57 ± 0.15
1 MAPP	1030 ± 31.2	26.1 ± 0.8	9.5 ± 0.3	5.69 ± 0.35
1 MAPP/POE	1100 ± 18.6	25.2 ± 0.5	9.2 ± 1.3	31.1 ± 0.35
2	1030 ± 35.0	26.4 ± 0.1	9.9 ± 0.3	4.35 ± 0.05
2 MAPP	1110 ± 22.5	27.1 ± 0.1	9.5 ± 0.2	5.55 ± 0.05
2 MAPP/POE	848 ± 8.3	20.3 ± 0.1	9.7 ± 0.1	18.43 ± 2.3
3	1000 ± 10.5	23.7 ± 0.1	8.7 ± 0.1	3.1 ± 0.7
3 MAPP	844 ± 54.0	20.5 ± 1.5	10 ± 1.5	3.47 ± 0.6
3 MAPP/POE	842 ± 6.0	19.6 ± 0.5	9.5 ± 0.5	11.36 ± 3.5
cPP	787 ± 12.5	20.7 ± 0.5	10.0 ± 0.1	7.00 ± 0.8

**Table 5:** Mechanical properties of cast film based on foil recyclates.

Name	Tensile Modulus [MPa]	Tensile Strength [MPa]	Elongation at Break [%]
1	107.3 ± 37.1	12.4 ± 1.9	156.3 ± 43.5
1 MAPP	118.1 ± 45.0	24.6 ± 2.2	822.3 ± 68.5
1 MAPP/POE	141.8 ± 58.7	16.4 ± 1.8	792.0 ± 86,1
2	227.0 ± 67.5	25.1 ± 1.1	806.0 ± 21.6
2 MAPP	158.0 ± 60.8	22.4 ± 1.6	301.0 ± 53.4
2 MAPP/POE	182.8 ± 39.4	25.1 ± 4.8	822.8 ± 129.0
3	118.8 ± 47.1	13.7 ± 0.7	688.8 ± 27.7
3 MAPP	127.9 ± 68.2	14.4 ± 0.1	10.0 ± 0.8
3 MAPP/POE	76.1 ± 33.3	20.6 ± 3.0	836.2 ± 121.9
cPP	78.7 ± 256.0	24.9 ± 3.4	956.7 ± 28.3

**Table 6:** The results of the color change measurements expressed by CIE L*a*b* coordinates for injection-molded samples.

Name	L Lightness	a Green/+Red	b Blue/+Yellow
1	48.12	−0.65	1.04
1 MAPP	45.98	−0.73	1.66
1 MAPP/POE	46.89	−0.89	2.00
2	56.23	−0.76	0.75
2 MAPP	54.91	−0.94	0.97
2 MAPP/POE	54.76	−0.86	1.04
3	48.01	−1.62	−1.19
3 MAPP	49.82	−1.96	−1.98
3 MAPP/POE	51.58	−1.10	−2.07
cPP	33.21	−1.35	−2.74

**Table 7:** The results of the color change measurements expressed by CIE L*a*b* coordinates for foil samples.

Name	L Lightness	a Green/+Red	b Blue/+Yellow
1	47.32	−0.59	0.91
1 MAPP	46.50	−0.79	1.82
1 MAPP/POE	47.55	−0.81	2.18
2	55.99	−0,79	0.67
2 MAPP	55.37	−0.85	0.83
2 MAPP/POE	55,37	−0.85	0.89
3	47.92	−1.50	−1.2
3 MAPP	50.67	−1.78	−1.91
3 MAPP/POE	51.09	−1.20	−1.94
cPP	32.49	−1.20	−2.69

## Data Availability

Data are contained within the article.
